# Rapid loss of plastid *ndh* genes in slipper orchids (Cypripedioideae, Orchidaceae)

**DOI:** 10.3389/fpls.2025.1507415

**Published:** 2025-04-22

**Authors:** Victoria E. Ringelmann, Natascha D. Wagner

**Affiliations:** Department of Systematics, Biodiversity and Evolution of Plants (with Herbarium), University of Goettingen, Göttingen, Germany

**Keywords:** gene loss, ultrametric tree, plastome, phylogeny, *Paphiopedilum*, myco-heterotrophic lifestyle

## Abstract

**Introduction:**

The eleven plastid *ndh* genes encode for subunits of the *ndh* (NAD(P)H dehydrogenase-like) complex, which mediates electron flow in photosystem I. The loss of *ndh* genes in plants was observed in many different lineages of Viridiplantae. In lineages of Orchidaceae, the loss of *ndh* genes was often associated with myco-heterotrophy. However, in previous studies on this topic only a few slipper orchids were included. Our study aimed to analyze the loss of *ndh* genes within Cypripedioideae, a subfamily that is assumed to be fully autotroph.

**Methods:**

Based on a comprehensive sampling of 100 published plastomes representing 60% of Cypripedioideae species, the phylogenetic relationships were revealed on three levels. For family and subfamily levels, 57 and 66 plastid genes, respectively, were extracted and concatenated in Geneious, while for the genus-level phylogeny, complete plastomes were used to calculate a maximum likelihood tree. Additionally, divergence time estimates were performed to illuminate the evolutionary timeframe of the gene loss. The prevalence, pseudogenization and loss of *ndh* genes were assessed and visualized along the phylogenetic trees.

**Results:**

The results confirmed the four analyzed genera of Cypripedioideae to be monophyletic and could increase the resolution within the genera compared to previous studies. The diversification of the subfamily started at about 30 Ma with genus *Paphiopedilum* displaying the most recent diversification starting at about 11 Ma and showing most speciation events around 4 Ma. The rapid loss of plastid *ndh* genes within the subfamily Cypripedioideae, particularly in the genera *Mexipedium*, *Phragmipedium* and *Paphiopedilum* could be illustrated. Furthermore, the results illustrated that Cypripedioideae are in an early stage of plastid degradation.

**Discussion and conclusions:**

Recent studies showed that partial myco-heterotrophy (mixotrophy) is far more common in plant lineages than originally assumed. Based on our findings, we suggest that the possibility of a mixotrophic lifestyle within (sub-)tropical slipper orchids should be reevaluated. Further research regarding the reasons behind plastid gene loss in slipper orchids could provide a better understanding of the ecological evolution of Cypripedioideae.

## 1 Introduction

The plastome of land plants encodes for approximately 100 protein coding genes and 30-50 RNA genes, and is assumed to be highly conserved in gene content and order (Rivas et al., 2002). However, recent studies showed that plastomes might vary a lot regarding gene presence and gene order (Ruhlman and Jansen, 2021). Hu et al. (2022) indicated that the evolution of the plastid genome may be an adaptive response to abiotic stresses, i.e. temperature or water stress. The plastome contains highly conserved sequences and is utilized in a large number of phylogenetic studies, e.g., for further understanding of the molecular and adaptive evolution of Orchidaceae Juss (Guo et al., 2021). The eleven *ndh* genes encode for subunits of the NDH (NAD(P)H dehydrogenase-like) complex, which is important for the photosynthetic electron transport chain, and are present in all lineages of higher plants. Furthermore, *ndh* genes enable a better protection of the plant against environmental, i.e. oxidative, stress. Plastid *ndh* genes appear to have homologs in cyanobacteria, which is in accord with the endosymbiotic theory of the evolution of the chloroplast (Burrows, 1998). The NDH complex is one of the many adaptations of land plants to the challenges of populating terrestrial habitats, i.e. ultraviolet radiation, water limitation and drastic fluctuations in light intensity, but is now of low biological significance in extant plants (Ruhlman et al., 2015). Under favorable growth conditions, plants can grow without the NDH complex (Burrows, 1998; Ifuku et al., 2011). Furthermore, Martín et al. (2015) suggested that *ndh* genes could also be dispensable at low atmospheric CO2 concentrations. While the loss of plastid *ndh* genes was observed in various lineages, i.e. Geraniaceae Juss., Orchidaceae, Orobanchaceae Vent., Petrosaviaceae Hutch., Simmondsiaceae Tiegh. and Triuridaceae Gardner (Blazier et al., 2011; Lin et al., 2017; Kharabian-Masouleh et al., 2023), the majority of plants have all eleven plastid *ndh* genes. This may hint towards a selective advantage of a functioning NDH complex. The absence of *ndh* genes in many independent lineages shows the different possible reasons and functions, e.g., in Cactaceae *ndh* loss was observed and it was suggested to be related to CAM photosynthesis as an adaptation to dry habitats (Köhler et al., 2020). In other lineages, plants with missing or pseudogenized *ndh* genes tend to have a heterotrophic lifestyle in form of myco-heterotrophs or parasitism (Wicke et al., 2016; Lin et al., 2017). Such a correlation was also observed in Orchidaceae (Kim et al., 2015; Lin et al., 2017).

The family Orchidaceae comprises five subfamilies, namely Apostasioideae, Vanilloideae, Cypripedioideae, Orchidoideae and Epidendroideae. With over 25,000 species and approximately 800 genera, Orchidaceae are the second largest family of flowering plants and only exceeded by Asteraceae Bercht. & J.Presl (Cozzolino and Widmer, 2005; Chase et al., 2015). Since new orchid genera are being described each year, the phylogeny of orchids is far from being fully resolved (Chase et al., 2015; Pérez-Escobar et al., 2024). Orchids are not only distributed on five different continents, but their habitats and lifeforms vary greatly: there are orchid species which are terrestrial, lithophytic or, predominantly found in tropical areas, epiphytic; furthermore, myco-heterotrophic lifestyles can be found in nearly every subfamily (Kim et al., 2020). The loss of *ndh* genes is usually associated with a myco-heterotrophic lifestyle in Orchidaceae. Since many lineages of orchids contain pseudogenized or lost *ndh* genes, the individual lineages experienced independent gene losses (Wicke et al., 2016; Kim et al., 2020). Additionally, in orchid species that lacked all *ndh* genes, the chlororespiration function was lost (Kim et al., 2015). So far, the prevalence of *ndh* genes has been primarily investigated in Orchidoideae and Epidendroideae, which are the two largest subfamilies of Orchidaceae, while the other subfamilies like Cypripedioideae were not subject to such comprehensive studies or were represented by a very small and restricted sampling (Kim et al., 2020).

**Figure 1:**
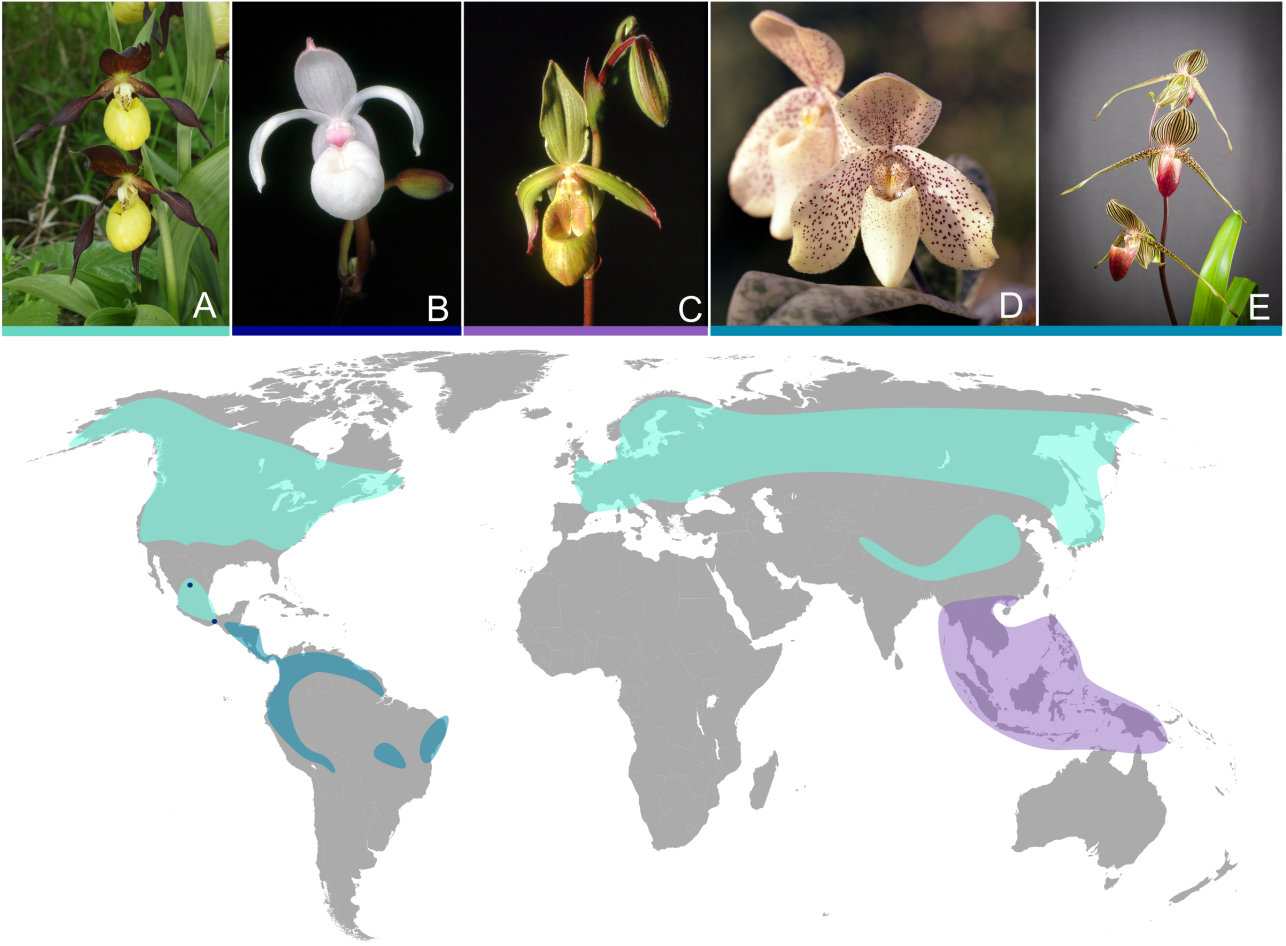
Five species of the subfamily Cypripedioideae. **(A)** *Cypripedium calceolus*, **(B)** *Mexipedium xerophyticum*, **(C)** *Phragmipedium lindleyanum*, **(D)** *Paphiopedilum concolor* and **(E)** *Paphiopedilum rothschildianum*. The map shows the distribution ranges of genera *Cypripedium* (light blue), *Mexipedium* (dark blue dots), *Phragmipedium* (petrol) and *Paphiopedilum* (purple). For the distribution ranges of the genera of Cypripedioideae we utilized the GBIF database. Orchid pictures **(A-E)** were taken from Wikimedia and fall under figure licenses (Krisp, 2013; Orchi, 2005; Orchi, 2006a; Orchi, 2006b; Takebayashi, 2018).

The slipper orchids (Cypripedioideae) contain five monophyletic genera (*Cypripedium* L., *Mexipedium* V.A.Albert & M.W.Chase, *Paphiopedilum* Pfitzer, *Phragmipedium* Rolfe and *Selenipedium* Rchb.f.) and 169 species, of which 136 occur in the Old World and 33 in the New World (Chase et al., 2015; Pérez-Escobar et al., 2024). The name slipper orchid is derived from one of their distinct morphological synapomorphies, the pouch-like labellum, which resembles a slipper (**Figure 1**). Further morphological synapomorphies regarding the flower are a shield-like staminode, two fertile stamens, and united lateral sepals or a synsepa (Cox et al., 1997). The temperate species of slipper orchids are terrestrial, while most (sub-)tropical species grow as epiphytes or lithophytes. Cypripedioideae occupy diverse habitats and are successfully adapted to different environments. Based on a recent study using target sequencing data, Cypripedioideae diversified about 30 Ma and where once wide spread in the boreotropics, while they show today an ancient relictual range (Pérez-Escobar et al., 2024). The genus *Paphiopedilum* is regarded as the most recent diversifying genus within slipper orchids (5-7 Ma; Tsai et al., 2020). The ancestor of the slipper orchids likely had a continuous distribution in the boreotropics followed by migration southwards to both sides of the Pacific Ocean due to the climatic cooling in the late Cenozoic (Guo et al., 2012; Pérez-Escobar et al., 2024). The current distribution of *Cypripedium* differs highly from the other genera of slipper orchids (**Figure 1**). *Cypripedium* is distributed in temperate and subtropical habitats of the northern hemisphere, with a main distribution in alpine areas, where it is exposed to high light intensity and rarely suffers from water stress (Brieger and Schlechter, 1992; Guo et al., 2012; Hu et al., 2022). *Phragmipedium* and the monotypical genus *Mexipedium* can be found in tropical Central and South American habitats (Guo et al., 2012).

*Paphiopedilum* is distributed in tropical areas of South East Asia from India eastward to the Philippean and Suda Isles and southward to New Guinea and the Solomon Isles (Brieger and Schlechter, 1992). *Paphiopedilum* species grow terrestrial, as lithophytes or as epiphytes and were because of its ornamental value subject to several recent studies (Tsai et al., 2020; Hu et al., 2022; Liu et al., 2022). The strongly differing habitats of slipper orchids and a distribution around multiple contintents makes them an ideal group to study the adaptive evolution of plastomes, specifically the loss of *ndh* genes.

The loss of *ndh* genes within Orchidaceae has been the topic of numerous scientific studies, but so far Cypripedioideae were only sparsely sampled (Kim et al., 2020). This study aims to gain further insight into the loss of plastid *ndh* genes within the subfamily, which will help to understand general mechanisms of plastid gene loss, to develop ideas on compensatory strategies to survive without an NDH complex and to increase our knowledge on evolution and adaptation of slipper orchids to different habitats. We want to analyze whether the gene loss happened gradually or if one or multiple sudden gene losses occurred during the evolutionary history of the subfamily. Futhermore, if the loss of *ndh* genes happened multiple times independently and lastly, we want to assess the gene loss in a temporal time frame.

## 2 Material and methods

### 2.1 Sampling

The 163 plastomes used in this study were derived from NCBI Genbank. In detail, three plastomes of Vanilloideae, 101 of Cypripedioideae, 15 of Orchidoideae and 43 of Epidendroideae. The plastomes were downloaded in the GenBank (full) format. Additionally, one sample of *Allium cepa* and one sample of *Iris sanguinea* were included as outgroups. Only one sample per species was selected, since an interspecific variation of *ndh* genes seemed very unlikely. Additionally, the large majority of Cypripedioideae species in the NCBI database were represented by only one sample and using consistently one sample per species avoided sampling bias. For Vanilloideae, at least one sample for each of the two tribes was selected. The samples represented approximately 60% of Cypripedioideae species. Three of the four Orchidaceae tribes and twelve of 16 Epidendroideae tribes were represented. A full list of samples and their reference names can be found in **Supplementary Material** (**Supplementary Table S1**).

### 2.2 Gene extraction

Since the plastome showed high variation between the different species of Orchidaceae and Cypripedioideae regarding plastome length as well as order, length and prevalence of plastid genes, a subset of genes was extracted for the phylogenetic analysis. The respective genes were chosen because of their presence in at least 90% of samples of either Orchidaceae or Cypripedioideae. This way we received well aligned genes and avoided too many gaps. The resulting subset for Orchidaceae consisted of 57 plastome genes (**Supplementary Table S2.1**) from 85 samples (three Vanilloideae, 22 Cypripedioideae, 16 Orchidoideae and 42 Epidendroideae plus two outgroup samples). For the phylogenetic analysis of subfamily Cypripedioideae, 66 genes were extracted from the plastome samples (**Supplementary Table S2.2**). These were in detail three Vanilloideae as outgroup taxa and 101 Cypripedioideae samples representing eleven *Cypripedium*, one *Mexipedium*, six *Phragmipedium* and 83 *Paphiopedilum* species. Individual plastid genes were extracted from the full plastomes with Geneious 2023.2 (Kearse et al., 2012). The genes were aligned separately with MAFFT v7 (Katoh and Standley, 2013), implemented in Geneious 2023.2, using the FFT-NS-2 algorithm. For a better alignment process, poorly aligned sections were removed manually within Geneious 2023.2. Then the genes were aligned a second time with MAFFT v7, this time using the L-INS-i algorithm. Afterwards, the gene alignments were concatenated to one supermatrix with Geneious 2023.2.

On family and subfamily level the plastome show many structural differences and huge inversions or rearrangements, therefore the alignment of full plastomes was impossible. However, on genus level within subfamily Cypripedioideae the plastome structure was conserved and thus it was possible to use complete plastomes for genus *Paphiopedilum* to increase the phylogenetic signal for this closely related group. The 83 samples were completed with three *Phragmipedium* samples to root the tree, that is *Ph. longifolium* Rolfe, *Ph. lindenii* Dressler & N.H.Williams and *Ph. kovachii* J.T.Atwood, Dalström & Ric.Fernández. All 86 plastomes were aligned with MAFFT v7. The calculation was done on the High Performance Cluster (HPC) of the GWDG (Gesellschaft fuer wissenschaftliche Datenverarbeitung Göttingen), Göttingen.

### 2.3 Phylogenetic analysis and ultrametric tree calculation

To analyze the relationships of species and the evolutionary history of the *ndh* genes, we performed maximum likelihood analyses for all plastid gene alignments (Orchidaceae, Cypripedioideae) and the full plastome alignment of *Paphiopedilum*. The maximum likelihood (ML) trees were calculated with RAxML 8.2.12 (Stamatakis, 2014) with the maximum likelihood model GRT-GAMMA. 100 rapid bootstrap replicates were calculated, followed by a ML search. The resulting trees were visualized with FigTree 1.4.4 (Rambaut, 2014).

The dated phylogeny was based on the dataset for Cypripedioideae. We roughly followed the settings of Pérez-Escobar et al. (2021). BEAUTI v.2.6 (Bouckaert et al., 2014) was used to set a GTR nucleotide substitution model with Γ distribution rate with four categories. We used an uncorrelated log-normal strict molecular clock in combination with a prior clock rate interval of 0.0001–0.001 substitutions/site/Ma, modelled by a uniform distribution. A birth–death tree process, modelled by a uniform distribution for the birth and relative death rates was used. For the secondary calibration we set the most recent common ancestor (MRCA) prior to 30Ma for the crown of Cypripedioideae following previously published dated phylogenies on Orchidaceae (Zhang et al., 2023; Pérez-Escobar et al., 2024), modelled by normal distributions (σ = 1). We run 10 million generations, sampling every 1 000 generation. Finally, we used TREEANNOTATOR v.2.6 (https://www.beast2.org/treeannotator/) with a burn-in threshold of 10% to compute a maximum credibility consensus tree from the Markov chain Monte Carlo (MCMC) posterior trees.

### 2.4 *ndh* gene assessment

The plastomes downloaded from Genbank were fully annotated. The prevalence of *ndh* genes was assessed with Geneious 2023.2 based on the existing gene annotations from Genbank entries. Since the length of genes varied a lot within Orchidaceae, we defined an internal reference gene length for the *ndh* genes based on the Cypripedioideae samples. The gene annotations were used to determine what gene length should be set as reference gene length for comparison. A gene length was set as reference gene length if the exact number of bp was present in at least 50% of Cypripedioideae samples, which was the case for *ndh*B, *ndh*C, *ndh*E, *ndh*G, *ndh*H, *ndh*I and *ndh*J. For the *ndh* genes with strongly varying lengths, namely *ndh*A, *ndh*D, *ndh*F and *ndh*K, the following procedure was used to determine the standard gene length: The average gene length was calculated two times. First, the average gene length was calculated based on all Cypripedioideae samples that had the gene annotated. The second calculation excluded samples that differed in their gene length by at least 30% from the first calculated average gene length. Finally, the second calculated average gene lengths were selected as reference gene length. Thus, the reference gene lengths were determined as following: 2,263 bp for *ndh*A, 2,242 bp for *ndh*B, 363 bp for *ndh*C, 1,513 bp for *ndh*D, 306 bp for *ndh*E, 2,118 bp for *ndh*F, 531 bp for *ndh*G, 1,182 bp for *ndh*H, 495 bp for *ndh*I, 477 bp for *ndh*J and 764 bp for *ndh*K. Afterwards, the actual presence of *ndh* genes in Cypripedioideae was determined manually by comparing the samples with up to three *Cypripedium* samples, namely *Cypripedium lichiangense* S.C.Chen & P.J.Cribb, *Cypripedium formosanum* Hayata and *Cypripedium calceolus* L. This was done to discover annotation errors in the Cypripedioideae samples. For the Orchidaceae and outgroup samples, the prevalence of *ndh* genes was based on the existing annotations of the samples and a correct annotation was assumed The *ndh* genes with a gene length below 80% or above 120% reference bp length were defined as pseudogenized. This included indels, sequence duplications and changes in reading frames. For Cypripedioideae, it was determined in more detail, if the pseudogenization resulted from truncation, i.e., that stop codons occurred within the annotated sequence, caused by mutations or frame shifts, resulting in (very) short functional reading frames. Genes with a length below 20% of the reference gene length or not found at all were defined as lost. The results of the gene assessment were visualized alongside the phylogenetic trees.

## 3 Results

### 3.1 Alignment statistics

The length of the alignment for the Orchidaceae family tree based on 85 samples and 57 plastid genes that passed our thresholds was 60,203 bp long including 24,995 SNPs (41.5%) and 13.2% missing data (incl. outgroup). For the Cypripedioideae the alignment of 66 genes resulted in 74,028 bp length, containing 12,067 SNPs (16.3%) and 11.24% missing data. Finally, the full plastome alignment for genus *Paphiopedilum* showed a length of 265,241 bp (150,136-163,948 bp), 35,289 SNPs (13.3%) and 40.34% missing data (incl. outgroups).

### 3.2 Phylogenetic trees and dated phylogeny

**Figure 2:**
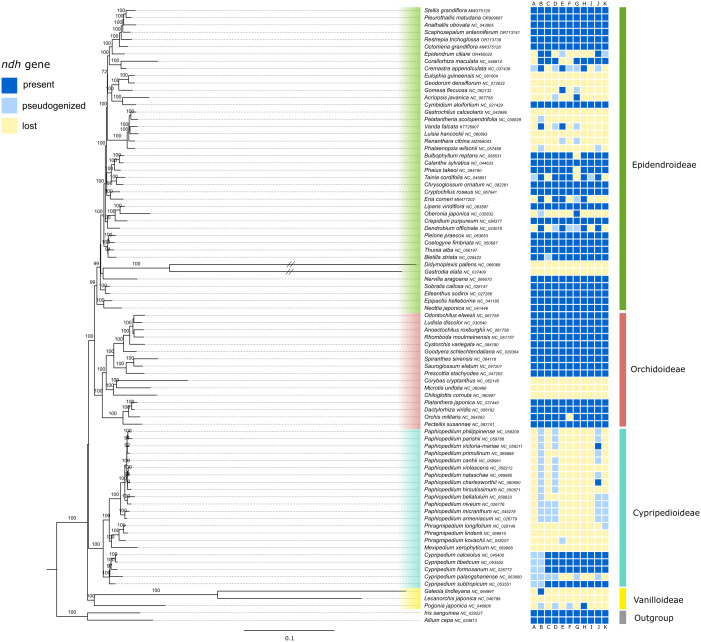
Phylogenetic relationships inferred from a RAxML analysis based on 57 plastid genes and 85 taxa of Orchidaceae. The tree was rooted with *Allium* and *Iris* used as outgroup. The four subfamilies were color-coded. The prevalence/loss of *ndh* genes is shown in the colored boxes next to taxon names following the legend in the upper left corner. Bootstrap values from 100 replicates are shown above branches. The branch length is based on the number of substitutions per site and then normalized (see RAxML manual).

The generated maximum likelihood tree represented four of the five subfamilies of Orchidaceae, i.e., Vanilloideae, Cypripedioideae, Orchidoideae and Epidendroideae (**Figure 2**, **Supplementary Figure S2** with full branch length). Three of the four subfamilies were highly supported as monophyletic in the ML tree, with a bootstrap support (BS) of 99-100%. Vanilloideae were the sister clade to the remaining three subfamilies. Cypripedioideae were shown to be in sister relationship with the clade containing Orchidoideae and Epidendroideae, however on an unsupported short branch. Within Cypripedioideae all four genera appeared monophyletic (BS=100%). The branches of the Gastroideae tribe (*Didymoplexis pallens* Griff. and *Gastrodia elata* Blume) were much longer than of all other Orchidaceae samples and therefore shortened in **Figure 2** (see **Supplementary Figure S2** with full branch length). The clades within Epidendroideae were poorly to well supported and the backbone of this subfamily showed very short branch lengths (**Figure 2**). Within Orchidoideae, clades were well supported (BS=100%). The majority of included tribes appeared to be monophyletic, except for Collabieae (*Calanthe sylvatica* (Thouars) Lindl., *Chrysoglossum ornatum* Blume, *Phaius takeoi* (Hayata) H.J.Su. and *Tainia cordifolia* Hook.f.), which was revealed as being polyphyletic.

**Figure 3:**
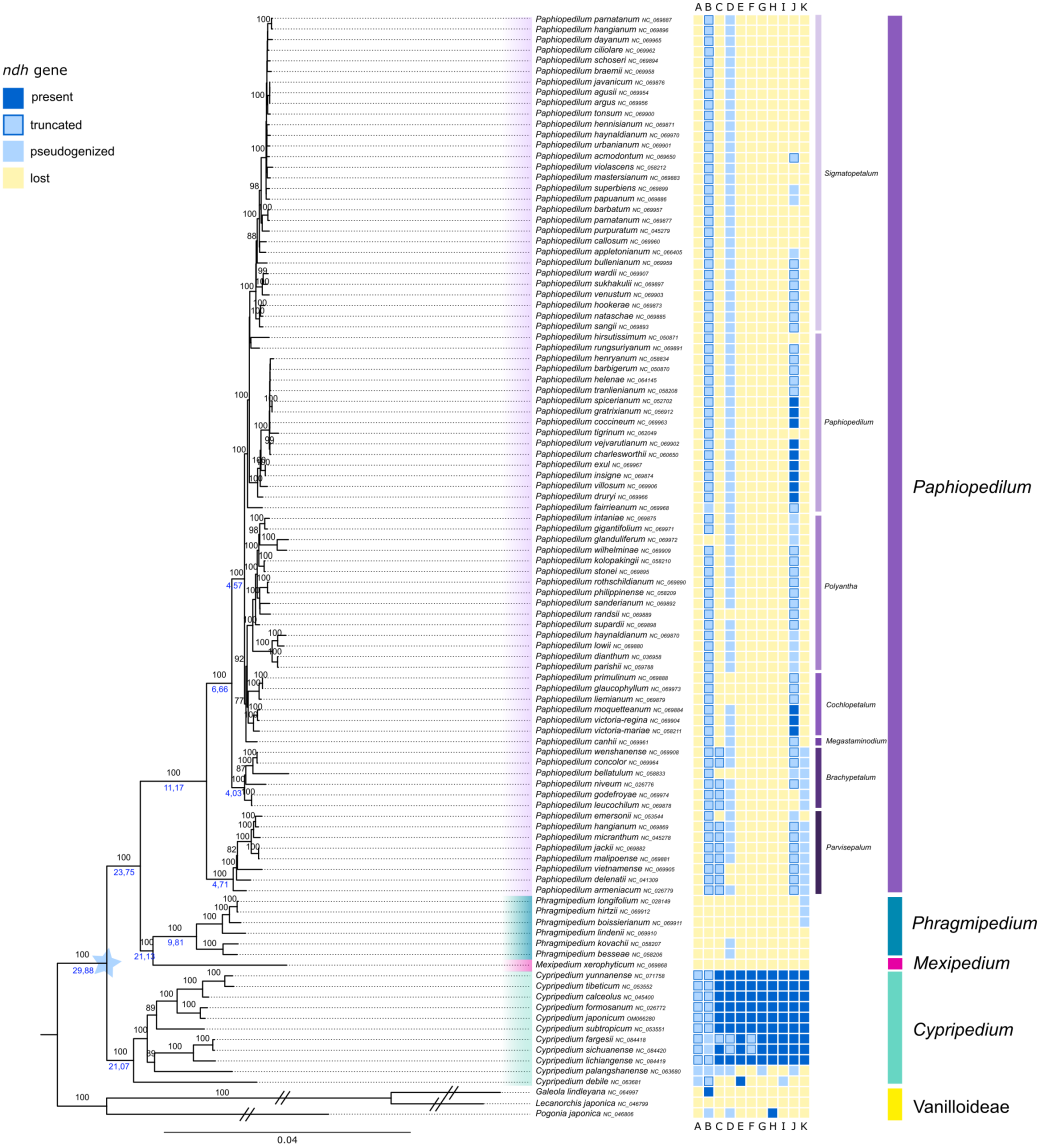
Phylogenetic relationships inferred from a RAxML analysis based on 66 plastid genes and 101 taxa of Cypripedioideae. The tree was rooted with three samples of Vanillioideae used as outgroup (branches shortened for illustration). The four genera were color-coded. The prevalence/loss of *ndh* genes is shown in the colored boxes next to taxon names following the legend in the upper left corner. Bootstrap values are shown above branches, node ages below selected branches (taken from BEAST analysis, **Supplementary Figure S1**). The branch length is based on the number of substitutions.

The generated ML tree of Cypripedioideae represents four of the five genera of the subfamily (*Cypripedium*, *Mexipedium*, *Phragmipedium* and *Paphiopedilum*, **Figure 3**, **Supplementary Figure S3**). The three sampled Vanilloideae species, *Pogonia japonica* Rchb.f., *Galeola lindleyana* Rchb.f. and *Lecanorchis japonica* Blume, were used as outgroup. The long branches were shortened in **Figure 3**, see **Supplementary Figure S3** for full branch length. The monophyly of the four slipper orchid genera was supported by very high bootstrap support values (BS=100%). *Cypripedium* was sister to the remaining genera, followed by a clade containing *Mexipedium* and *Phragmipedium* (BS=100%), to which *Paphiopedilum* was in sister position. Bootstrap values within *Cypripedium* were overall very high (BS=100%), with two exceptions of slightly lower bootstrap support (BS=89%). *Cypripedium debile* Rchb.f. was sister to these two clades. The genus *Paphiopedilum* was monophyletic, however, branch lengths within this genus in the ML tree of *Cypripedioideae* were very short (**Figure 3**). Therefore, we performed a phylogenetic tree based on full plastomes for this genus.

**Figure 4:**
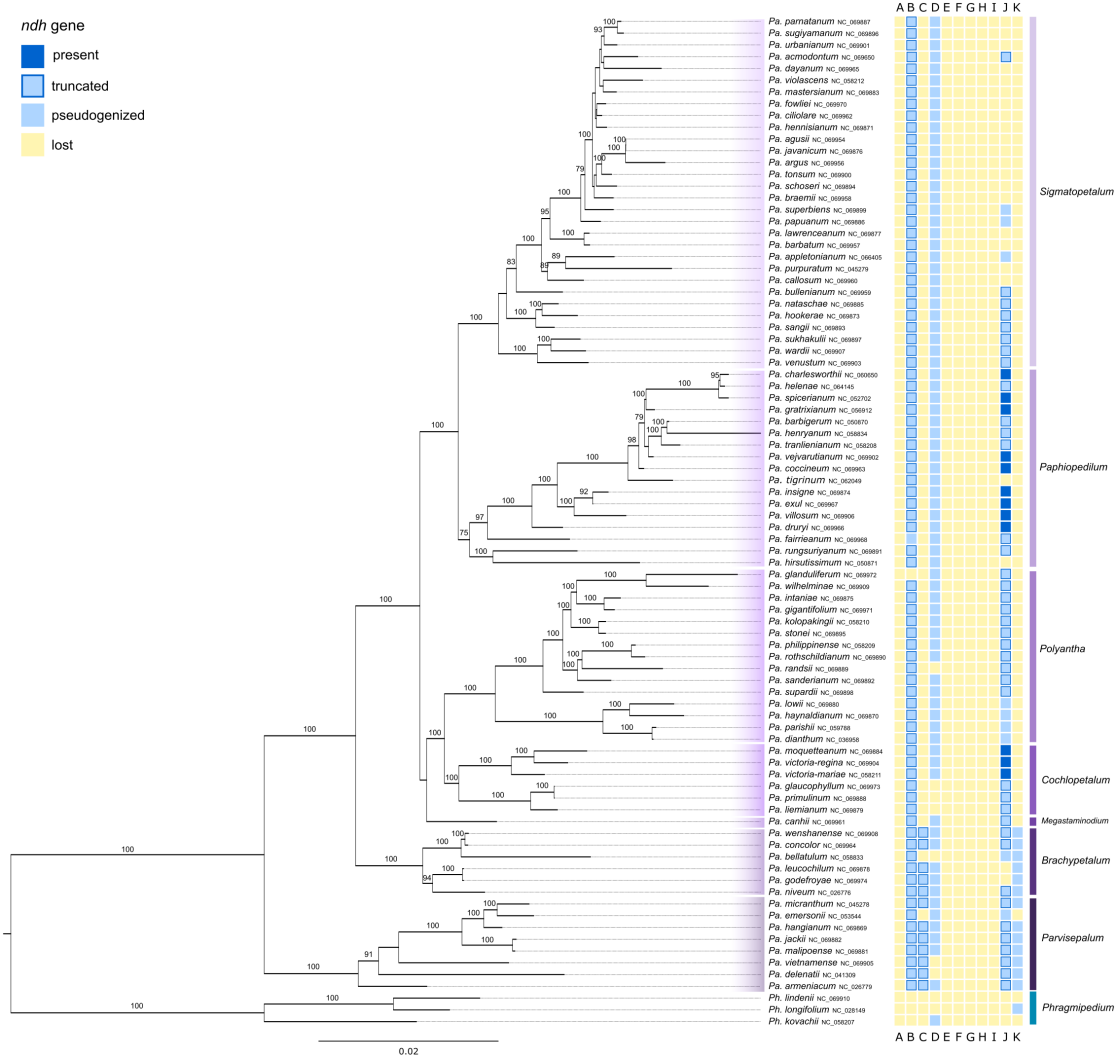
Phylogenetic relationships revealed from a RAxML analysis based on full plastomes of 85 taxa of *Paphiopedilum*. The tree was rooted with three samples of *Phragmipedium* used as outgroup. The subgenera were color-coded. The prevalence/loss of *ndh* genes is shown in the colored boxes next to taxon names following the legend in the upper left corner. Bootstrap values are shown above branches. The branch length is based on the number of substitutions.

The *Paphiopedilum* phylogeny based on full plastomes contained 83 species plus three outgroup taxa and showed well resolved clades (**Figure 4**). The seven subgenera of *Paphiopedilum* all appeared as monophyletic with a very high bootstrap support (BS=100%), the exceptions being subg.

*Paphiopedilum* with a slightly lower bootstrap support (BS=75%) and subg. *Megastaminodium* with low bootstrap support. Within subg. *Parvisepalum*, bootstrap values were very high (BS=91-100%) with only one clade being of lower support. Similar results can be observed in subg. *Brachypetalum*, where only one clade was of slightly lower but still good bootstrap support (BS=94%). Subg. *Megastaminodium*, subg. *Cochlopetalum* and subg. *Polyantha* formed one clade and each of the three subgenera was monophyletic with a varying bootstrap support. Within subg. *Cochlopetalum* all clades were highly supported (BS=100%). Subg. *Polyantha* was of very high bootstrap support (BS=100%). The clade containing subg. *Paphiopedilum* and subg. *Sigmatopetalum* had a very high bootstrap support as well (BS=100%). Subg. *Paphiopedilum* was well supported (BS=75-100%). Subg. *Sigmatopetalum* showed variable bootstrap values, ranging between low support and 100% BS.

The full results of the dated phylogeny of the Cypripedioideae using BEAST are shown in **Supplementary Figure S1** and the node ages for the main clade were also illustrated in **Figure 3**. The subfamily started to diverge about 30 Ma. The split of *Mexipedium, Phragmipedium* and *Paphiopedilum* happened about 24 Ma. The genus *Phragmipedium* showed a crown age of about 10 Ma, while genus *Paphiopedilum* diversified 11 Ma. Within the latter genus, the clade containing the five subgenera *Megastaminodium, Cochlopetalum, Polyantha, Paphiopedilum* and *Sigmatopetalum* started to diverge at about 4.5 Ma.

### 3.3 *ndh* gene assessment

Within the family Orchidaceae, the loss of *ndh* genes can be observed in all four represented subfamilies (**Figure 2**). While the two outgroup samples contain all eleven plastid *ndh* genes, the loss of *ndh* genes was observed in all three samples of Vanilloideae. Within Orchidoideae, the three samples of Diurideae have lost all eleven plastid *ndh* genes, while many of the other sampled species still contain all genes. Within the subfamily Epidendroideae, only the tribes Neottieae, Sobralieae and Nervilieae showed the presence of all *ndh* genes. However, several species lost part or all *ndh* genes, especially in tribes Cymbidieae, Gastroideae and Vandeae. Overall, there was a strong variation in the degradation of plastid *ndh* genes, as can be seen in **Figure 2**.

Within the slipper orchids, the majority of *ndh* genes were lost at least twice. *Cypripedium* was the only genus that retained most of the eleven *ndh* genes in the majority of sampled species. Both *Cypripedium debile* Rchb.f. and *Cypripedium palangshanense* Tang & F.T.Wang lost numerous *ndh* genes, with the remaining ones, except for *ndh*E in *C. debile*, being pseudogenized. The genes *ndh*A and *ndh*B were pseudogenized in every *Cypripedium* sample.

Within the clade containing *Mexipedium* and *Phragmipedium*, all *ndh* genes were lost in the majority of samples. The gene *ndh*D was found pseudogenized in two *Phragmipedium* samples and lost in the remaining samples. Three other samples of *Phragmipedium* contained functional fractions of the *ndh*K gene (**Figure 3**). All *Paphiopedilum* samples have lost the *ndh*A, *ndh*E, *ndh*F, *ndh*G, *ndh*H and *ndh*I genes. The gene *ndh*B was truncated in the majority of samples within this genus. *Ndh*C was truncated in subg. *Parvisepalum* and subg. *Brachypetalum*, and lost in the other subgenera. The gene *ndh*D was pseudogenized in the majority of *Paphiopedilum* samples. Seven samples did not have a functional *ndh*D. *Ndh*J was functional in twelve samples of this genus (within subg. *Paphiopedilum* and subg. *Cochlopetalum*), truncated in 39 samples and pseudogenized or lost in the remaining samples (**Figure 3**). Only the samples within subg. *Parvisepalum* and subg. *Brachypetalum* contained a pseudogenized *ndh*K, the remaining samples have lost this gene. The better resolved phylogeny based on full plastomes revealed the same pattern (**Figure 4**).

## 4 Discussion

In our study we aimed to analyze the *ndh* gene loss in members of the subfamily Cypripedioideae and put it in an evolutionary frame work. One sample per species was selected for the calculations of the phylogenetic trees. Previous studies showed slight variation in the retention of *ndh* genes between different samples of one species within Orchidaceae, but not within Cypripedioideae (Kim et al., 2020; Hu et al., 2022).

### 4.1 Phylogenetic relationships and ndh gene loss in Orchidaceae

The phylogenetic analysis of Orchidaceae was based on 91 samples representing four of the five subfamilies and was based on 64 plastid genes (**Supplementary Table S2**). The phylogenetic relationships of the subfamilies and tribes of Orchidaceae were congruent with the consensus phylogeny of Orchidaceae presented in Chase et al. (2015) and a recent phylogeny based on more than 600 nuclear genes (Zhang et al., 2023). The long branches of *Didymoplexis pallens* and *Gastrodia elata*, both Epidendroideae tribe Gastrodieae, resulted from the drastically reduced plastome size (35.304 bp & 51.241 bp) and thus missing data, probably due to their myco-heterotrophic lifestyle (Leake, 1994).

The presence of *ndh* genes within Orchidaceae was similar to Kim et al. (2020), except that their study included more samples of myco-heterotrophic orchids and thus showed more variation in *ndh* gene presence within Cranichideae and Neottieae. In contrast to Kim et al. (2020), our study conducted a more representative tree of Orchidaceae by including samples of multiple genera belonging to a tribe (**Figure 2**), while Kim et al. (2020) represented multiple tribes by only one genus, namely Diurideae, Nervillieae, Gastrodieae and Collabieae. Given the diversity of species within a genus, the inclusion of several samples is necessary to draw a more complete picture of *ndh* gene loss in Orchidaceae. Lin et al. (2017) suggested that the loss of *ndh* genes is related or possibly even necessary for the heterotrophic phase orchids have to go through at their early life stages. This does seem rather unlikely, as the *ndh* genes have been shown to be present in many independent orchid lineages (**Figure 2**; Kim et al., 2020).

### 4.2 Phylogenetic relationships and ndh gene loss in slipper orchids

The phylogenetic tree of Cypripedioideae was based on 66 plastid genes and 101 samples of slipper orchids, representing four out of the five genera (**Figure 3**). The phylogenetic relationships have strong similarities with previous phylogenetic studies of Cypripedioideae (Cox et al., 1997; Tsai et al., 2020; Guo et al., 2021; Szlachetko et al., 2021; Hu et al., 2022). The results of the BEAST analysis used a crown age of 30 Ma for Cypripedioideae (Zhang et al., 2023; Pérez-Escobar et al., 2024). The split of the clade containing *Phragmipedium* and *Mexipedium*, and *Paphiopedilum* happened 24 Ma. The latter genus started diversifying 11 Ma, which is younger than with former studies based on plastid data (Guo et al., 2012), however, similar to more recent studies on Orchidaceae evolution (Pérez-Escobar et al., 2024). We are aware that the similar diversification times might be the result of the calibration and clock model we applied following Pérez-Escobar et al. (2024). Like in the *Paphiopedilum* phylogenies based on plastid protein coding genes (Tsai et al., 2020; Hu et al., 2022), many clades of subg. *Sigmatopetalum* were of low support. The branches of the genus *Paphiopedilum* were relatively short, which suggests a recent radiation of this genus (Tsai et al., 2020), which is in accordance with the recent divergence of this subgenus in our study (c. 3 Ma). It may even be a hint that the *Paphiopedilum* genus is in its preliminary stage of speciation (Guo et al., 2021). Over the decades, the phylogeny of *Paphiopedilum* has repeatedly been proven to be difficult to resolve (Cox et al., 1997). Additionally, phylogenetic studies based on plastids do not provide enough genetic variation to study closely related species (Provan et al., 2001). Furthermore, reticulate evolution and clonal propagation, which are not uncommon within this rather young genus, can have yet unknown effects on the plastome genome evolution (Guo et al., 2021). However, a recent study on *Paphiopedilum* based on 41 full plastomes resolved the relationships of this genus (Liu et al., 2022). We followed their example and to increase resolution within the genus, we complemented our study with a genus-level tree for *Paphiopedilum* based on full plastomes (**Figure 4**). The phylogenetic relationships of the *Paphiopedilum* subgenera were in accord with previous phylogenetic studies of this genus (Cox et al., 1997; Guo et al., 2012; Tsai et al., 2020; Hu et al., 2022; Liu et al., 2022). However, here we included a comprehensive sampling of 83 species and the resolution and bootstrap support of *Paphiopedilum* subgenera were much higher than in previous studies with similar sample sizes regarding the phylogeny of *Paphiopedilum* or *Cypripedioideae* (Cox et al., 1997; Hu et al., 2022; Liu et al., 2022).

Our results regarding the loss of *ndh* genes within Cypripedioideae (**Figure 3**) showed that most *ndh* genes were found functional in *Cypripedium*, but were either lost or pseudogenized in the genera *Mexipedium*, *Phragmipedium* and *Paphiopedilum*, which confirms previous findings (Kim et al., 2020; Guo et al., 2021; Hu et al., 2022). The loss of *ndh* genes was not gradually as expected, but presumably happened rapidly and at least twice. First, in the early-branching lineages of *Cypripedium*, namely in *C. palangshanense* and *C. debile*, and second, in the common ancestor of *Mexipedium*, *Phragmipedium* and *Paphiopedilum* about 24 Ma. The pattern of gene loss in *Paphiopedilum* in comparison to *Phragmipedium* and *Mexipedium* differs in the presence of the genes *ndh* B, C and J (**Figure 3**), indicating that more than two events of *ndh* losses occurred more recently during the evolution of slipper orchids, i.e., 21 and 11 Ma, respectively. In a recent study on the evolution of epiphytism in Orchidaceae, Zhang et al. (2023) stated a shift of terrestrial to litophytic and epiphytic lifestyle within Cypripedioideae. While *Cypripedium* is exclusively terrestrial, *Mexipedium* is regarded as lithophytic and the genera *Phragmipedium* and *Paphiopedilum* contain also epiphytic species. The genera *Phragmipedium* and *Mexipedium* occur in the New World and diversified 21 Ma, however, the crown age of *Phragmipedium* is much younger (9.8Ma). The loss of all *ndh* genes might be the result of the shift to the subtropical environment. The transition in lifestyle could also be an explanation for the rapid loss of *ndh* genes within the subfamily. In this case we would assume to find a pattern of lifestyle and *ndh* gene loss in our study, i.e., that epiphytic species lost more genes than non-epiphytic ones. However, within *Paphiopedilum* and *Phragmipedium*, we do not see such a lifestyle related pattern of *ndh* loss. Therefore, the lifestyle is no exclusive explanation for the rapid loss of many *ndh* genes in both genera. Liu et al. (2022) stated that the *ndh* gene variations were found in IR (inverted repeat) boundaries in *Paphiopedilum* species and thus were considered to be attributed to the recombination of IRs. Next, they speculated that the degradation of *ndh* genes in *Paphiopedilum* was the result of adaptive evolution to a low light environment. Species of *Paphiopedilum* living beneath dense canopies with insufficient light and exhibited the characteristics of shade plants (Liu et al., 2022).

A large majority of Cypripedioideae retained an intact copy of *ndh*B, *ndh*D and *ndh*J. This observation hints toward a possible higher importance of these specific genes and should be material for further study, which could bring new insights regarding the functions of these genes. Horváth et al. (2000) showed that tobacco plants with a defective *ndh*B gene were inhibited in their photosynthesis when growing under low air humidity, leading to a possible explanation for why the selection toward an intact *ndh*B gene was observed. However, *Paphiopedilum* is only distributed in tropical habitats, in which low air humidity is not a defining characteristic. Still, environmental factors and the adaptation to them are probably an important driving force in the evolution of the plastome within slipper orchids (Hu et al., 2022). This theory is further supported by the major difference in *ndh* degradation between *Cypripedium* and the other three genera analyzed in this study (**Figure 3**). The habitats and distribution ranges between these two clades are distinctly different from each other (**Figure 1**). The loss of *ndh* genes does infer with a plants` adaptability to changing environmental factors like sudden changes in light intensity or strong fluctuations of temperature (Yamori et al., 2011; Martín et al., 2015). The habitats of tropical species of slipper orchids provide much less fluctuations in temperature and light intensity, whereas the temperate montane habitats of *Cypripedium* usually provide stronger fluctuations of environmental factors. This may be a reason why the *ndh* genes have been retained in the majority of the analyzed *Cypripedium* samples. Additionally, a correlation between the loss of *ndh* genes and a natural distribution in shady habitats has also been observed in other genera, i.e. *Allium* L (Scobeyeva et al., 2021). Thus, adaptations to different light and temperature conditions might play a role in the degradation or retention of *ndh* genes.

Kim et al. (2015) theorized that the *ndh* genes may have been transferred to the nuclear genome. However, it is known that orchids that lost their plastid *ndh* genes have lost their nuclear *ndh* genes in most cases as well (Lin et al., 2017). Although a study conducted by Lin et al. (2015) found that fragments of chloroplast genes have been transferred to the mitochondrial genome, these genes do not get translated into functional proteins. However, no samples of Cypripedioideae were used in the study conducted by Lin et al. (2017), and the orchid species represented in their study were not fully autotroph. In a study on *Paphiopedilum*, the authors found fragments of *ndh* genes transferred to the nucleus and assumed that gene transfer in *Paphiopedilum* might be explained by the decreased demands on photosynthesis and plastid translational capacities due to the adaptation to the unique habitat of the species – without giving any further explanation (Liu et al., 2022). Further studies are needed to analyze the possible transfer of plastid *ndh* genes into the nuclear genome within the autotrophic Cypripedioideae, but so far it is assumed the NDH complex loses its function without functioning plastid *ndh* genes. Our study also showed that the majority of *ndh* genes were either lost or pseudogenized in the genus *Paphiopedilum*, which makes it unlikely that the species of this genus have a functioning NDH complex. Physiological characteristics suggested a non-functioning NDH complex; when compared to *Cypripedium* leaves, *Paphiopedilum* leaves had a significantly lower photosynthetic capacity (Guan et al., 2011; Yang et al., 2018). These results may correlate with the loss of *ndh* genes, as has been shown in Lin et al. (2017). Thus, the lower photosynthetic capacity in *Paphiopedilum* can be seen as further proof of the loss of a functioning NDH complex. Since the NDH complex is not only important for photosynthesis but also in the protection of the plant against environmental stress, it is of interest to observe whether *Paphiopedilum* shows inferred abilities regarding these functions as well, or if there are any yet unknown factors compensating for it. According to Wicke et al. (2016), the loss of plastid *ndh* genes is the first step of gene loss associated with photosynthetic genes and congruent with the transition to a heterotrophic lifestyle. The loss of *ndh* genes within Cypripedioideae observed in this study could hence be a sign of a heterotrophic lifestyle within this subfamily. However, current research states no signs of a heterotrophic lifestyle within any Cypripedioideae species (Guo et al., 2021). The cultivation of myco-heterotrophic orchids has repeatedly been proven to be challenging (Brieger and Schlechter, 1992), but Cypripedioideae species and cultivars are regularly sold as outdoor and indoor plants. If Cypripedioideae had an obligate myco-heterotrophic lifestyle, they would not be such a common ornamental plant due to the difficulty of cultivation. This does not necessarily mean that all Cypripedioideae are solely autotrophic plants. Wicke et al. (2016) observed that some photosynthetic parasites (hemiparasites) can grow without a host plant and thus are considered to be facultative parasites. This may also be the case for the slipper orchid genera *Mexipedium*, *Phragmipedium* and *Paphiopedilum*. Some myco-heterotrophic plants obtain the majority of carbon from photosynthesis and only a small portion from mycorrhizal fungi, often to compensate for low photosynthetic rates resulting from shady habitats; this is also called mixotrophy (Selosse and Roy, 2009). The previously named genera of slipper orchids are known to grow in shady habitats, which make a myco-heterotrophic lifestyle within these genera more likely. Merckx and Freudenstein (2010) also suggested that myco-heterotrophy and partial myco-heterotrophy may have been an adaptation to shady grounds of forests as an alternative way to collect carbon. Furthermore, a recent study conducted by Tian et al. (2023) regarding the soil fungal community structures of *Paphiopedilum* subg. *Brachypetalum* found major differences between the habitats of each species. This indicates a high specificity regarding mycorrhizal partners in *Paphiopedilum*, which is often observed in myco-heterotrophic plants. However, the prospect of mixotrophic or hemiparasitic plants is not much studied, although recent studies showed that mixotrophy may be far more widespread among plants than originally assumed, i.e. in Ranunculaceae Juss (Giesemann et al., 2020).

Based on previous findings and the results from this study, we suggest reevaluating the possibility of a mixotrophic or facultative myco-heterotrophic lifestyle for Cypripedioideae, particularly in *Mexipedium*, *Phragmipedium* and *Paphiopedilum*. This theory should be tested in future studies to gain more understanding of the ecology of slipper orchids, along with research regarding their habitats and ecological adaptation. By understanding how and with which mechanisms slipper orchids adapted to their habitats and what genetic changes lead to vulnerabilities regarding environmental stressors, the conservation of this orchid subfamily will become feasible. Further, such studies will help to increase our understanding regarding the plants reaction to a changing climate.

## Data Availability

The original contributions presented in the study are included in the article/**Supplementary Material**. Further inquiries can be directed to the corresponding author.
