# Supplementary material for: Rapid loss of plastid ndh genes in slipper orchids (Cypripedioideae, Orchidaceae)
Source: Front Plant Sci. 2025 Apr 22;16:1507415. doi: 10.3389/fpls.2025.1507415 (PMC12053501; doi:10.3389/fpls.2025.1507415)
Supplement: Supplementary file 1 [file DataSheet1.pdf]

Ringelmann et al. 2024 - Supplement Material Figure S1

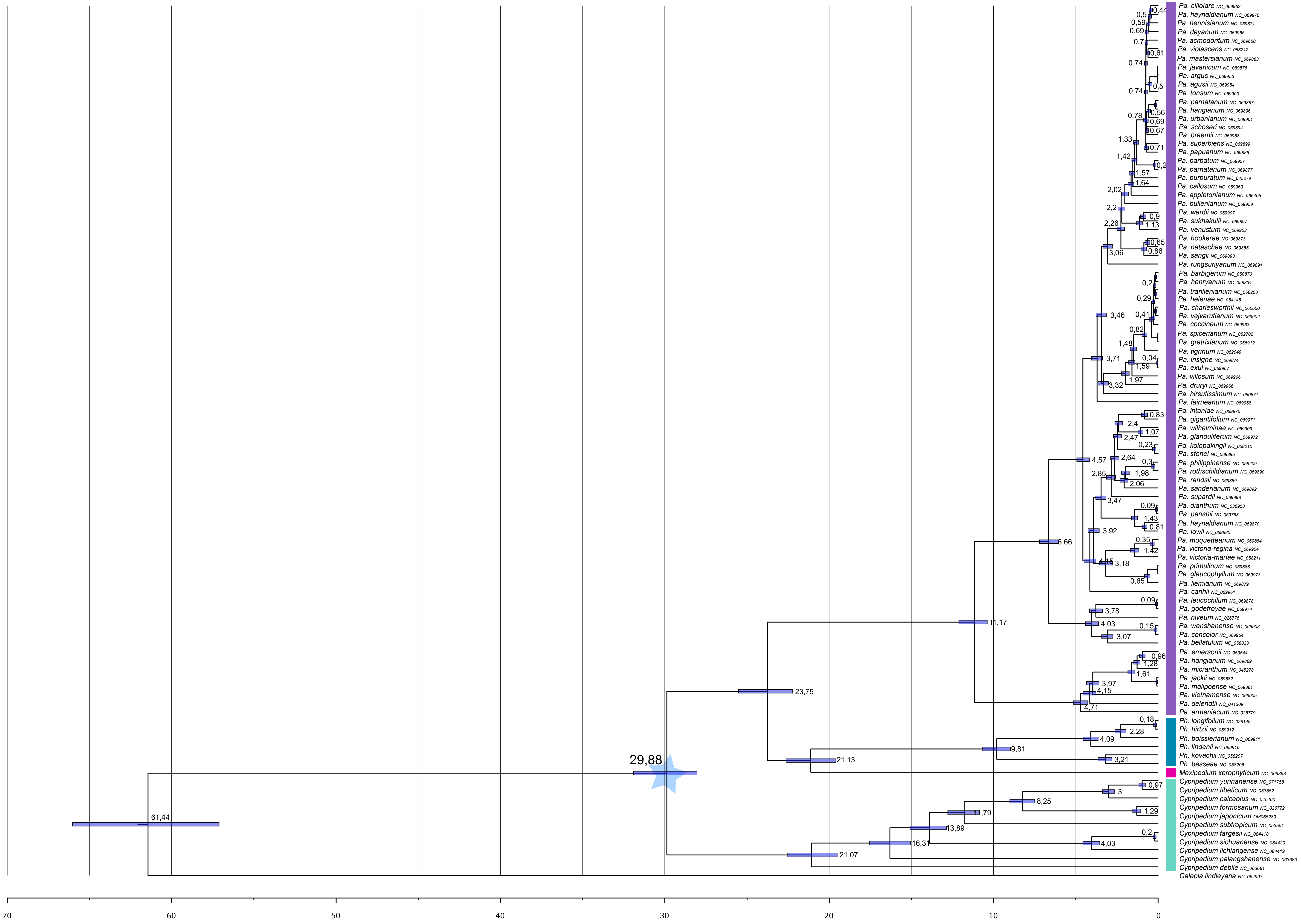

Supplement Figure S1. Ultrametric tree resulted from a BEAST analyses based on the Cyrpredioideae dataset including 66 plastid loci and 104 taxa. The tree was calibrated at the node leading to Cypripedioideae. The respective genera are color-coded. For more details see Material & Methods.
