# Supplementary material for: Rapid loss of plastid ndh genes in slipper orchids (Cypripedioideae, Orchidaceae)
Source: Front Plant Sci. 2025 Apr 22;16:1507415. doi: 10.3389/fpls.2025.1507415 (PMC12053501; doi:10.3389/fpls.2025.1507415)
Supplement: Supplementary file 2 [file DataSheet2.pdf]

Ringelmann et al. (2024) Supplement Figure S2

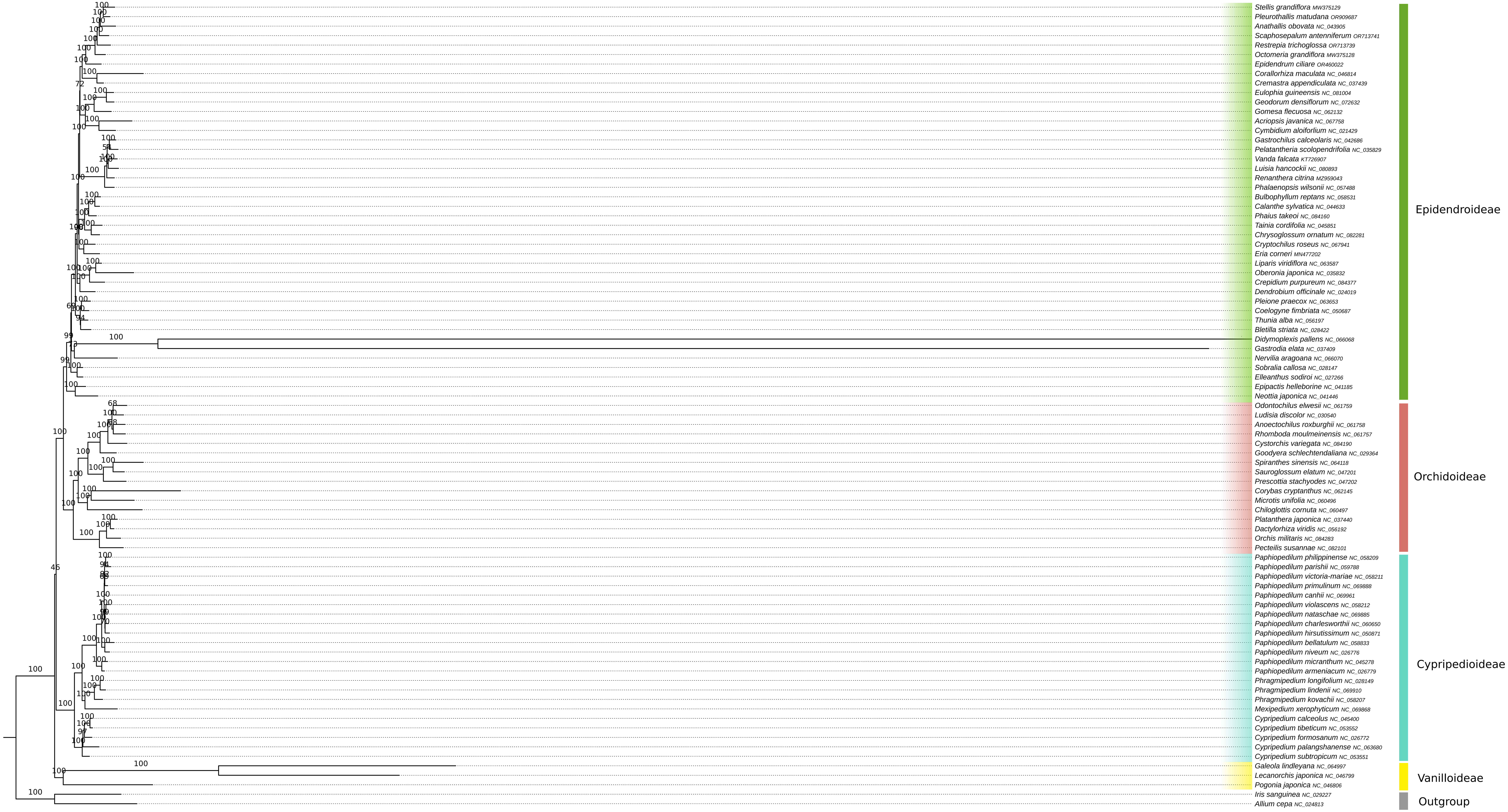

Supplement Figure S2. Phylogenetic relationships revealed from a RAxML analyses based on 57 plastid genes and 85 taxa of Orchidaceae. The tree was rooted with Allium and Iris used as outgroup. The four subfamilies were color-coded. Bootstrap values are shown above branches.
