# Supplementary material for: Rapid loss of plastid ndh genes in slipper orchids (Cypripedioideae, Orchidaceae)
Source: Front Plant Sci. 2025 Apr 22;16:1507415. doi: 10.3389/fpls.2025.1507415 (PMC12053501; doi:10.3389/fpls.2025.1507415)
Supplement: Supplementary file 3 [file DataSheet3.pdf]

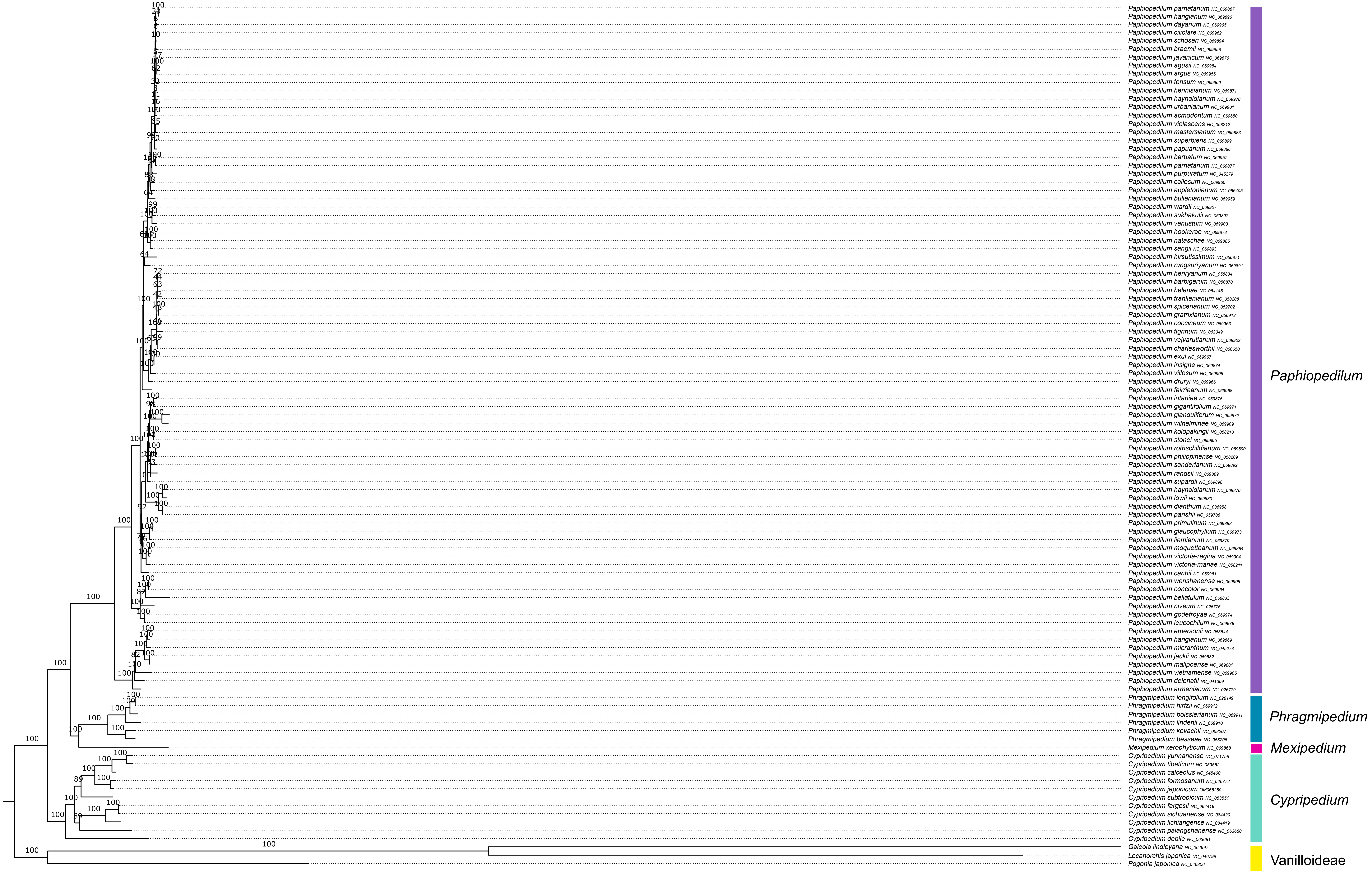

Supplement Figure S3. RAxML phylogeny based on 101 taxa of Cypripedioideae and three Vanillioideae as outgroup. Bootstrap values are shown above branches.
