# Supplementary material for: Rapid loss of plastid ndh genes in slipper orchids (Cypripedioideae, Orchidaceae)
Source: Front Plant Sci. 2025 Apr 22;16:1507415. doi: 10.3389/fpls.2025.1507415 (PMC12053501; doi:10.3389/fpls.2025.1507415)
Supplement: Supplementary file 5 [file Table2.pdf]

Supplement Table S2

Table S 2.1.: List of extracted genes for the phylogenetic tree of Orchidaceae. Genes marked with (*) were modified.

accD*	atpI	psaB*	psbC	psbJ	rbcL	rpl23	rps8	rps18*	atpA*	ccsA	petD*	psbD
psbK	rpl2*	rpl32	rpoC2*	rps11	rps19*	atpB	clpP*	petG	psaI	psbE	psbL	rpl14
rpl33	rps2*	rps12*	ycf1*	atpE	infA*	petL	psaJ*	psbM*	rpl16*	rpl36	rps3	rps14
ycf2*	atpF*	matK*	petN	psbA*	psbT	rpl20*	rpoA*	rps4	rps15	atpH	petA	psaA*
psbB	psbI	psbZ*	rpl22*	rps7	rps16*							

Table S 2.2: List of extracted genes for the phylogenetic tree of Cypripedioideae. Genes marked with (*) were modified.

accD*	atpI	petB	psaB	psbD	psbK*	rbcL*	rpl23	rpoC1	rps8	rps18*	atpA	ccsA
petD*	psaI	psbE	psbL	rpl2	rpl32*	rpoC2*	rps11	rps19	atpB	clpP*	petG	psaJ
psbF	psbM	rpl14	rpl33	rps2*	rps12*	ycf1*	atpE	infA	petL	psbA*	psbH*	psbN
rpl16*	rpl36	rps3	rps14	ycf2*	atpF*	matK*	petN	psbB	psbI	psbT	rpl20*	rpoA*
rps4	rps15	ycf3*	atpH	petA	psaA	psbC	psbJ	psbZ*	rpl22*	rpoB*	rps7	rps16*
ycf4												
